# Sonographic Detection of Cutaneous Myiasis

**DOI:** 10.5811/cpcem.2019.7.43250

**Published:** 2019-09-18

**Authors:** Elizabeth Nicholas, Kevin Gaskin, Susan Wojcik

**Affiliations:** Upstate Medical University, Department of Emergency Medicine, Syracuse, New York

## Abstract

Cutaneous maggots are occasionally encountered in the emergency department. We present a case in which maggots were visually identified and ultrasound was used to detect additional maggots below the skin crevices in a patient with elephantiasis nostras verrucosa.

## CASE PRESENTATION

A 47-year-old male with super morbid obesity, bilateral lower extremity dermatitis and lymphedema, presented to our emergency department with redness, pain, and potential maggots in the left lower extremity. One year prior he had burned his left leg with boiling water but never sought medical treatment. On exam, he had chronic skin changes of venous stasis, lymphedema, and burn scarring. The skin was lichenified with deep crevices (Image). This condition, seen in severe chronic lymphedema, is called elephantiasis nostras verrucosa.

Two maggots were identified on the skin surface. Ultrasound with linear transducer was used to determine whether or not gas was present in the tissues. Rather than identify gas in the tissues, sonography revealed additional maggots within the crevices of the skin. These appeared as oblong-shaped structures with a hypoechoic rim and a hyperechoic center. This structure is similar to criteria for the presence of myiasis suggested by Bouer et al.^1^ In this case, some of the larvae borrowed beneath the folds of the patient’s skin and would intermittently “pop-up” toward the ultrasound probe (Video). The maggots were removed with forceps, and the wounds were thoroughly cleansed and disinfected. There remained concern for bacterial superinfection; therefore, the patient was admitted to the medicine service, and an infectious disease consult was obtained. Patient received seven days of intravenous piperacillin-tazobactam and was discharged home on an additional seven days of oral amoxicillin clavulanate.

## DISCUSSION

Myiasis is infection with fly larvae, usually occurring in tropical or subtropical areas. Accidental myiasis (also called pseudomyiasis) is caused by flies that have no preference or need to develop in a host but do so on occasion.^2^ Transmission in this case occurred through the deposit of eggs on the patient’s lichenified skin surface. The larvae of the housefly then feed on both live and necrotic tissue causing myiasis to develop. Myiasis is often misdiagnosed because its symptoms are non-specific. Clues include travel to an endemic area (which our patient did not have), one or more non-healing lesions on the skin, pruritis, movement under the skin, or pain.^3^ While this patient did have a few scattered maggots on his legs, ultrasound was able to readily reveal the presence of more maggots below the most superficial skin surface. Mechanical removal of fly larvae is usually curative unless bacterial superinfection has occurred, in which case antibiotics would be indicated.

**CPC-EM Capsule**

What do we already know about this clinical entity?

*Cutaneous myiasis is occasionally encountered in the emergency department. Most of the time the diagnosis is immediately visible to the examiner*.

What is the major impact of the image(s)?

*This case of a patient with elephantiasis nostra verrucosa illustrates the ability of the examiner to use ultrasound to identify additional maggots which were not readily visible on physical exam*.

How might this improve emergency medicine practice?

*Ultrasound may be able to detect myiasis in other chronic skin conditions where maggots may not be immediately visible to the examiner*.

## Supplementary Information

Video. Note the maggot on the left of the image (arrow) which briefly appears above the skin surface and subsequently burrows beneath the skin surface.

## Figures and Tables

**Image:**
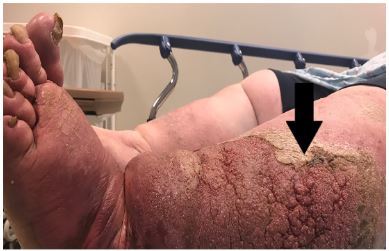
A photograph of the patient’s left lower extremity showing lichenfication of the skin (arrow) and crevices.
